# Detection of DNA of *Babesia canis* in tissues of laboratory rodents following oral inoculation with infected ticks

**DOI:** 10.1186/s13071-020-04051-z

**Published:** 2020-04-03

**Authors:** Alexandra Corduneanu, Teodor Dan Ursache, Marian Taulescu, Bogdan Sevastre, David Modrý, Andrei Daniel Mihalca

**Affiliations:** 1 Department of Parasitology and Parasitic Diseases, University of Agricultural Sciences and Veterinary Medicine Cluj-Napoca, Cluj-Napoca, Romania; 2 Department of Pathology, University of Agricultural Sciences and Veterinary Medicine Cluj-Napoca, Cluj-Napoca, Romania; 3 Department of Physiopathology, University of Agricultural Sciences and Veterinary Medicine Cluj-Napoca, Cluj-Napoca, Romania; 4 CEITEC-VFU, University of Veterinary and Pharmaceutical Sciences, Brno, Czech Republic; 5 Department of Pathology and Parasitology, University of Veterinary and Pharmaceutical Sciences, Brno, Czech Republic; 6 Biology Centre, Institute of Parasitology, Czech Academy of Sciences, České Budějovice, Czech Republic

**Keywords:** *Babesia canis*, *Dermacentor reticulatus*, Mouse, Gerbil, Oral inoculation

## Abstract

**Background:**

*Babesia* spp. are apicomplexan parasites which infect a wide range of mammalian hosts. Historically, most *Babesia* species were described based on the assumed host specificity and morphological features of the intraerythrocytic stages. New DNA-based approaches challenge the traditional species concept and host specificity in *Babesia*. Using such tools, the presence of *Babesia* DNA was reported in non-specific mammalian hosts, including *B. canis* in feces and tissues of insectivorous bats, opening questions on alternative transmission routes. The aim of the present study was to evaluate if *B. canis* DNA can be detected in tissues of laboratory rodents following oral inoculation with infected ticks.

**Methods:**

Seventy-five questing adult *Dermacentor reticulatus* ticks were longitudinally cut in two halves and pooled. Each pool consisted of halves of 5 ticks, resulting in two analogous sets. One pool set (*n* = 15) served for DNA extraction, while the other set (*n* = 15) was used for oral inoculation of experimental animals (*Mus musculus*, line CD-1 and *Meriones unguiculatus*). Blood was collected three times during the experiment (before the inoculation, at 14 days post-inoculation and at 30 days post-inoculation). All animals were euthanized 30 days post-inoculation. At necropsy, half of the heart, lung, liver, spleen and kidneys were collected from each animal. The presence of *Babesia* DNA targeting the *18S* rRNA gene was evaluated from blood and tissues samples. For histopathology, the other halves of the tissues were used. Stained blood smears were used for the light microscopy detection of *Babesia*.

**Results:**

From the 15 pools of *D. reticulatus* used for the oral inoculation, six were PCR-positive for *B. canis*. DNA of *B. canis* was detected in blood and tissues of 33.3% of the animals (4 out of 12) inoculated with a *B. canis-*positive pool. No *Babesia* DNA was detected in the other 18 animals which received *B. canis*-negative tick pools. No *Babesia* was detected during the histological examination and all blood smears were microscopically negative.

**Conclusions:**

Our findings demonstrate that *B. canis* DNA can be detected in tissues of mammalian hosts following ingestion of infected ticks and opens the question of alternative transmission routes for piroplasms.
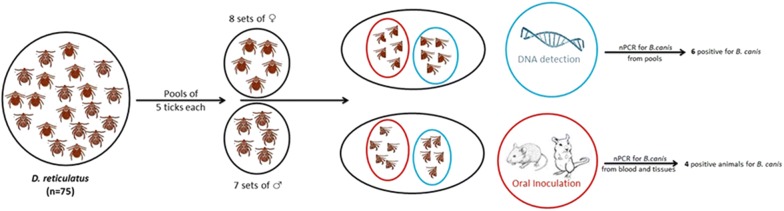

## Background

*Babesia* spp. are apicomplexan parasites which infect a wide range of mammalian hosts and often cause important clinical disease in domestic animals, and occasionally in wildlife and humans [1–4]. All *Babesia* spp. for which the life-cycle is known are transmitted by ticks during blood-feeding [5, 6].

Historically, *Babesia* species were described based on the assumed host specificity and morphology of the intraerythrocytic stages [7, 8]. Nevertheless, new molecular approaches do challenge the traditional species concept and host specificity of *Babesia* spp. [9]. Using such tools, the presence of *Babesia* DNA was reported in mammalian hosts that were not previously known to be susceptible to the infection [8]. Piroplasms ‘specific’ to horses (*B. caballi* and *Theileria equi*) were found in the blood of dogs from Croatia and Romania [10, 11]. *Babesia canis* and *Babesia vulpes* (reported as *T. annae* and *Babesia microti*-like piroplasm), considered to be canid-specific piroplasms (reported in wolves, red foxes, golden jackals) were identified in cats from Portugal [12, 13].

*Dermacentor reticulatus*, the only known vector of *B. canis* [14–16] is a relatively generalist tick. Although the immature stages of *D. reticulatus* feed principally on rodents, *B. canis* has never been reported in this group of hosts. Recent studies reported *B. canis* DNA in the feces, heart tissues, and engorged ticks (*Ixodes simplex*, *I. vespertilionis*) of European bats [17–19]. These findings might suggest alternative routes of transmission, such as oral ingestion of infected *D. reticulatus* ticks [17]. Such routes are well known for other tick-borne haemoprotozoans, such as *Hepatozoon canis* or *H. americanum* which are transmitted to dogs following ingestion of infected *Rhipicephalus* [20], *Haemaphysalis* [21] and *Amblyomma* [22–24] ticks. Apicomplexans of the genus *Hemolivia* are also known to be transmitted *via* tick ingestion to their vertebrate hosts [25–28]. These routes of transmission seem to represent evolutionary adaptations related to the feeding habits of the vertebrate hosts [29]. Carnivores are likely ingesting ticks when they feed on their prey [30, 31], while tortoises ingest ticks when feeding on vegetation [25]. Hence, such a route could be also the case for the repeated findings of *B. canis* DNA in bats. In this context, the aim of our study was to evaluate if oral ingestion of ticks infected with *B. canis* results into presence of detectable DNA in tissues of otherwise unexpected hosts.

## Methods

### Ticks

**Fig. 1:**
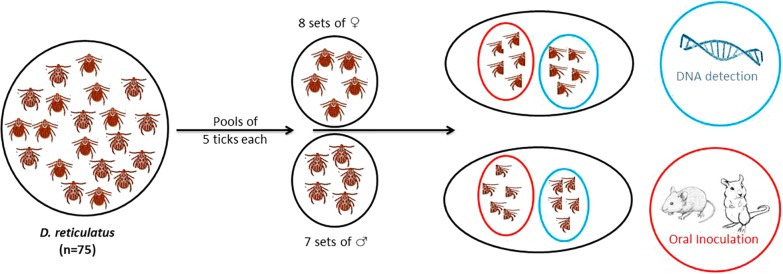
Schematic representation of experimental protocol

In May 2018, a total of 109 ticks were collected from Lazuri (47° 53′ 15″ N, 22° 53′ 30″ E), Satu-Mare County, Romania, by flagging. The ticks were kept in an aerated large plastic tube, in which a small piece of wet cotton was placed to maintain humidity until further processing. Morphological identification was performed individually for each tick using morphological keys [32]. Only the ticks identified as adult *Dermacentor reticulatus* (*n* = 75; 40 females and 35 males) were further used for the experimental trials. The 75 *D. reticulatus* ticks were divided in 15 pools, each containing 5 ticks of the same sex (8 pools of females and 7 pools of males). Each tick was longitudinally cut in half, while still alive. One pool set (15 pools) was used for DNA extraction, while the other set of pools (15 pools) was used for oral inoculation of experimental animals (Fig. 1).

### *Experimental* animals and study design

The total number of animals used in this study was 32 (16 adult mice, *Mus musculus* line CD-1 and 16 adult *Meriones unguiculatus*). They were housed individually in commercial plastic cages. The animals were maintained in standard conditions. Each tick pool was triturated together with a drop of physiological saline. From the obtained suspension, half the volume was inoculated orally to a mouse (*n *= 15) and the other half to a gerbil (*n* = 15), using a plastic single-use pipette. Additionally, one mouse and one gerbil were used as uninfected negative controls and inoculated only with physiological saline.

From both mice and gerbils blood was collected at three time points (30 μl was collected from each animal at each collection time): (i) before the inoculation of tick triturate in order to exclude the presence of piroplasms (molecular assay described below); (ii) at 14 days post-inoculation (pi); and (iii) at 30 days pi. At the first two collections, the blood was collected from both, mice and gerbils, by lateral tail vein puncture after light sedation with isoflurane. At the third collection blood was collected from the retro-orbital sinus, also under light isoflurane sedation. Each blood sample was mixed with PBS buffer (170 μl) and kept at 4 °C until DNA extraction. A small drop of blood from each sample at each sampling time was used for smears.

After the oral inoculation, each animal was clinically evaluated daily for a period of 30 days. At 30 days pi, the animals were euthanized by prolonged narcosis with isoflurane. The necropsy was performed, and from each animal half of the heart and spleen, a lobe of lung and liver, and one kidney were collected for DNA isolation; the other parts of these organs were fixed in 10% formalin for histopathological examination.

### Molecular assays

Genomic DNA from ticks and tissues was isolated using a commercial kit (Isolate II Genomic DNA Kit; Bioline, London, UK) according to the manufacturer’s instructions. Nested PCR amplifications targeting the *18S* rDNA gene (561 bp) were performed using two sets of primer pairs [33, 34]. The amplification profile used was described previously [35]. DNA isolated from the blood of a dog from Romania which was naturally infected with *B. canis* was used as a positive control. The sample was confirmed to be positive for *Babesia* spp. using the same protocols [33, 34] followed by sequencing. A DNA-free water was used as a negative control. PCR products were visualized by electrophoresis in a 1.5% agarose gel stained with RedSafe™ 20,000× nucleic acid staining solution (Chembio, St Albans, UK). Their molecular weight was assessed by comparison with a molecular marker (Hyperladder IV; Bioline). PCR-positive amplicons were purified using Isolate II PCR and Gel Kit (Bioline) and sequenced (Macrogen Europe, Amsterdam, Netherlands). The sequences were compared to those available in GenBank using the Basic Local Alignment Search Tool (BLAST) analysis (BLASTn algorithm).

### Haematological and histological examination

Two smears made from blood collected at 14 and 30 days pi from each experimental animal were Giemsa-stained and examined. At least 100 fields were examined under immersion oil objective (100× magnification) before the sample was considered free of piroplasms. All *B. canis* PCR-positive tissues were used for histopathological examination. The samples were routinely processed, embedded in paraffin wax, cut into 3–4 µm sections, and stained with haematoxylin and eosin (H&E). For each positive sample, two slides were examined using an Olympus BX51 microscope. The photomicrographs were taken using an Olympus SP 350 digital camera and Olympus stream image-analysis software (Olympus Corporation, Tokyo, Japan).

## Results

The health condition of the animals did not change for the entire period of the study.

### Molecular analyses

From the total number of 15 pools of *D. reticulatus* used for the oral inoculation of mice and gerbils, six were found positive for *B. canis* DNA (Table 1). In experimental animals, *B. canis* DNA was identified in blood and tissues of four animals (Table 1). *Babesia canis* DNA was not detected in tissues of animals which received non-infected tick pool suspension. Control animals were also PCR-negative. All the smears collected from the experimental animals were microscopically negative for the presence of *Babesia* spp.

**Table 1:** Presence of *Babesia* sp. DNA in tick pools, blood and tissues

Host	Sample	Pool														
		P1	P2	P3	P4	P5	P6	P7	P8	P9	P10	P11	P12	P13	P14	P15
PCR tick		+	−	+	−	+	−	+	−	−	+	−	−	+	−	−
Mouse	B1	−	−	−	−	−	−	−	−	−	−	−	−	−	−	−
	B2	−	−	−	−	−	−	−	−	−	−	−	−	−	−	−
	H	−	−	−	−	−	−	−	−	−	−	−	−	−	−	−
	L	−	−	−	−	−	−	−	−	−	−	−	−	−	−	−
	S	−	−	−	−	−	−	−	−	−	−	−	−	−	−	−
	K	−	−	−	−	−	−	−	−	−	+	−	−	−	−	−
Gerbil	B1	−	−	−	−	+	−	−	−	−	+	−	−	−	−	−
	B2	−	−	−	−	+	−	−	−	−	−	−	−	+	−	−
	H	−	−	−	−	−	−	−	−	−	−	−	−	−	−	−
	L	−	−	−	−	−	−	−	−	−	+	−	−	−	−	−
	S	−	−	−	−	−	−	−	−	−	−	−	−	−	−	−
	K	−	−	−	−	−	−	−	−	−	−	−	−	−	−	−

*Abbreviations*: B1, blood collected at 14 days pi; B2, blood collected at 30 days pi; H, heart; L, liver; S, spleen; K, kidney

*Babesia canis* DNA was present in the blood collected at 14 days pi in two gerbils (P5 and P10) but at the end of the experiment (30 days pi) only the blood of one of these gerbils (P5) remained positive. In one gerbil (P13), the blood tested positive only at 30 days pi. At the tissue level, only the liver of one gerbil (P10) and the kidney of one mouse (P10) were PCR-positive for *B. canis*. All the smears collected from the experimental animals were microscopically negative for the presence of *Babesia* spp.

The sequences analysis revealed the presence of one genetic variant in all the 6 infected tick pools (GenBank: MK836022). The BLAST analysis of the positive sequences showed a similarity of 100% with several *B. canis* isolates from dog blood (e.g. GenBank: MK571831.1 and MK571830.1), ticks (e.g. GenBank: MK070118.1) and a golden jackal (e.g. GenBank: KX712122.1). The sequences of *B. canis* found in the blood and tissues of infected animals were all identical with the sequences found in the respective positive tick pool, but not identical with the positive control used.

### Histopathological examination

**Fig. 2:**
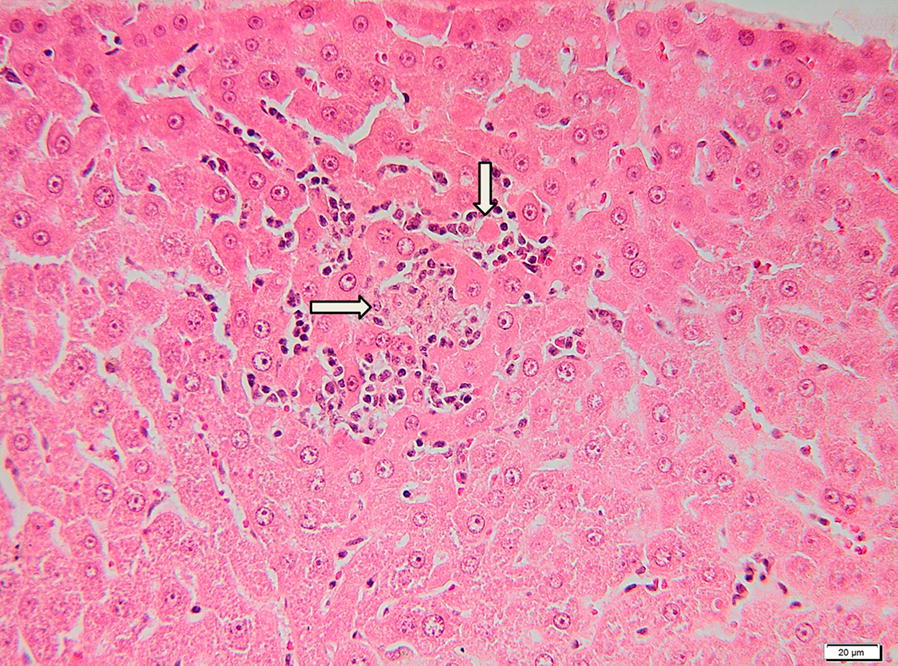
Histological section of the liver of a gerbil positive for *B. canis* DNA. The microphotograph is showing focal hepatic necrosis with neutrophils and macrophage infiltration (arrows). H&E staining. *Scale-bar*: 20 µm

**Fig. 3:**
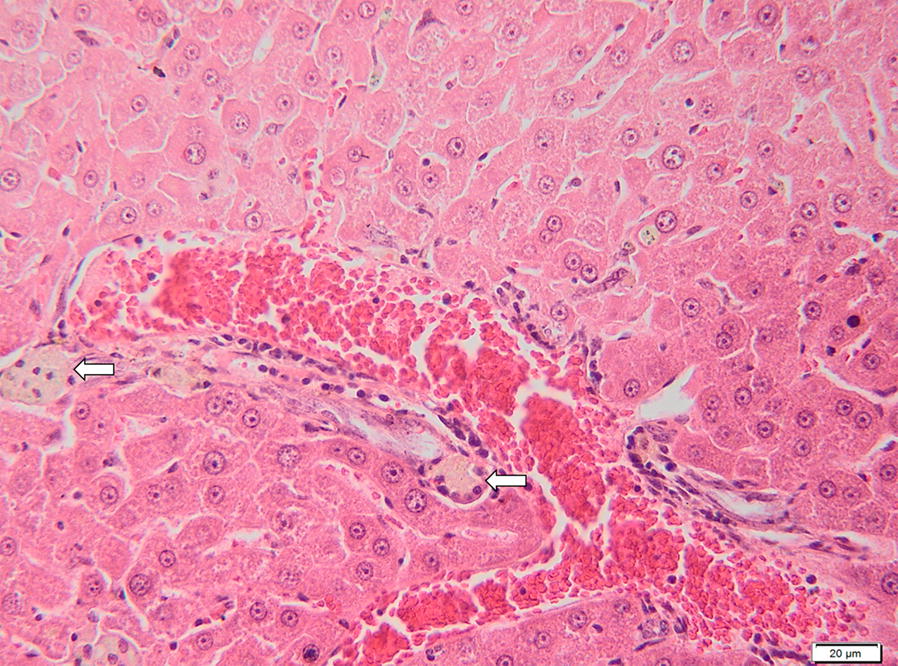
Liver of a gerbil positive for *B. canis* DNA. The image represents the portal tracts and sinusoids which presents individual and small groups of macrophages containing a fine granular yellow-brown pigment (hemosiderin) (arrows). H&E staining. *Scale-bar*: 20 µm

The hepatic parenchyma of the gerbil P5 showed diffuse congestion and randomly distributed foci of coagulative necrosis associated with small numbers of neutrophils and macrophages (Fig. 2). The portal areas were multifocally infiltrated with mononuclear cells dominated by lymphocytes, macrophages and few neutrophils. Both portal tracts and sinusoids showed individual and small groups of macrophages containing a finely granular yellow-brown pigment (hemosiderin) (Fig. 3).

**Fig. 4:**
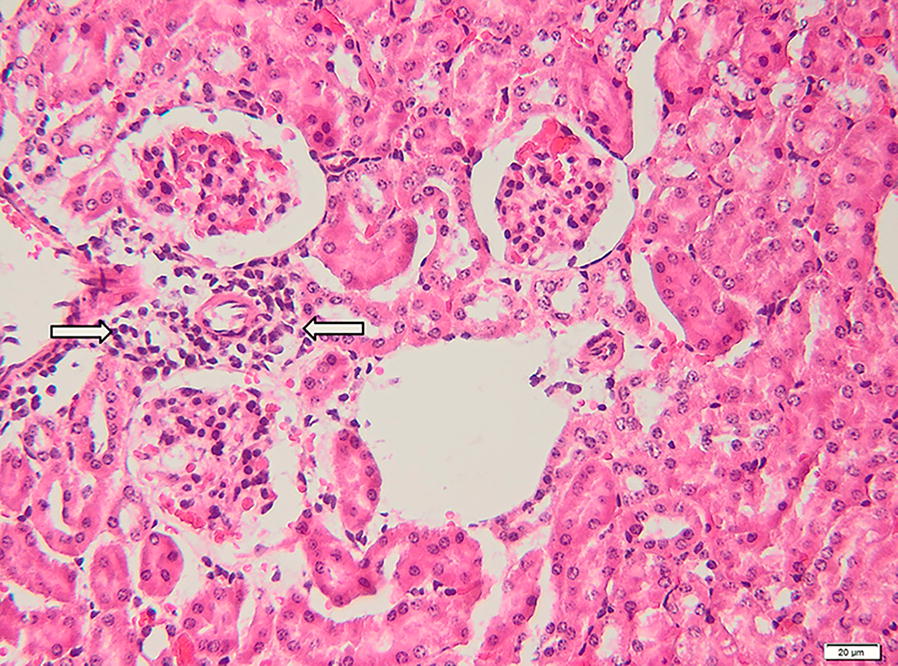
Kidney of a mouse positive for *B. canis* DNA. Microscopically, the perivascular areas and the tubule interstitium are mildly infiltrated with lymphocytes, neutrophils and macrophages (arrows). H&E staining. *Scale-bar*: 20 µm

**Fig. 5:**
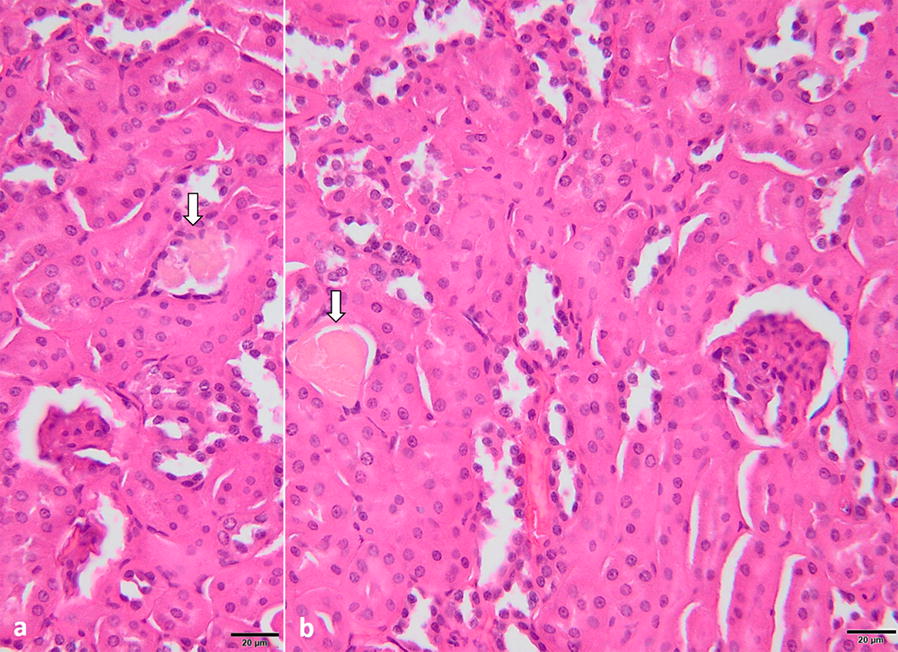
**a**, **b** Histological section of the kidney of a mouse positive for *B. canis* DNA. The microphotograph is showing area of hydropic degeneration and coagulative necrosis; the presence of haemoglobin casts can be also observed (arrows). H&E staining. *Scale-bar*: 20 µm

The renal parenchyma of the mouse P10 presented moderate renal congestion, particularly in the cortex; the interstitium was multifocally infiltrated by small numbers of mature lymphocytes, neutrophils and macrophages (Fig. 4). The renal proximal convoluted tubes were affected by vacuolar (hydropic) degeneration and coagulative necrosis: haemoglobin casts were occasionally found within the renal tubules (Fig. 5a, b). Mild glomerular hypercellularity was also observed in the positive cases.

## Discussion

This study presents experimental evidence for the presence of *B. canis* DNA in tissues of animals following the oral inoculation of *B. canis-*positive ticks. Although our study demonstrated that *Babesia* DNA can be found in tissues of animals after ingestion of infected ticks, this is not a proof of infection. The presence of the DNA does not imply the survival of the babesiae. To demonstrate this and the infection following oral inoculation of infected ticks, more detailed studies are probably needed, including the use of experimentally infected ticks. However, our study offers a possible explanation for the presence of *B. canis* DNA in the tissues of non-canid hosts, including the multiple reports in bats. As the use of bats for experimental studies is virtually impossible due to strict regulations, ethical issues, logistics and costs, we have designed our experiment using rodent models.

With more than 1200 species worldwide, bats’ diet is very diverse and depends on the geographical distribution of the bat species. All bats in Europe are insectivorous (except the Egyptian fruit bat) and belong to both orders, Yangochiroptera and Yinpterochiroptera [36]. Their diet may span many species, including flies, mosquitos, beetles, moths, crickets, grasshoppers, bees [37–40], but there are no reports of feeding on ticks. Interestingly, there are few recent reports of the presence of DNA of various piroplasmids and other hemoparasites of non-chiropteran hosts in samples collected from bats. Hornok et al. [17] reported the presence of *B. canis* DNA in feces of *Nyctalus noctula*, *Myotis alcathoe*, *Myotis daubentonii*, and *Pipistrellus pygmaeus* collected in Hungary. Corduneanu et al. [18] found DNA of *B. canis*, *B. gibsoni* and *Hepatozoon canis* in tissues of *N. noctula* and *P. pipistrellus* collected in the Czech Republic, Hungary and Romania. Additionally, Hornok et al. [19] reported the presence of several piroplasmid species in engorged bat-associated ticks (larvae of *I. vespertilionis*, and larvae, nymphs and females of *I. simplex*) collected from *M. daubentonii*, *M. dasycneme*, *Eptesicus serotinus*, *Miniopterus schreibersii* and *Rhinolophus hipposideros* in Hungary and Romania. All these reports are based on the detection of partial *18S* rDNA, which was considered as inconclusive by Uilenberg et al. [8], who recommended experimental transmission studies as more conclusive. As the known vectors of all these haemoparasites are ticks which do not feed on bats, the origin of this DNA remains uncertain, and raised the idea of the possibility of oral transmission.

Ingestion of ticks can occur either during grooming or predation of infested hosts in carnivores [31, 41] or accidentally from the vegetation in herbivores [25]. For some tick-borne apicomplexan parasites, the oral transmission with infected ticks is the main route of infection, as is the case of *Hepatozoon* spp. in carnivores [30] or *Hemolivia* spp. in tortoises [25]. However, in these cases, it is unclear how the infective stages disseminate from the intestine to the target tissues in the body (i.e. direct penetration of the gut or invasion of various hosts’ cells followed by blood migration) [24]. However, as yet, no tick DNA has been detected in bat feces [17], but no extensive studies have been completed.

Oral transmission of *Babesia microti* was demonstrated by Malagon & Tapia [42]. They infected mice by ingestion of blood and by cannibalism. The infection rate of mice which were orally inoculated with blood was 3.7% (5/135) and by cannibalism was 15.1% (12/79). The presence of parasites was detected on blood smears at the beginning of the experiment, at 7 days pi, followed by collection of blood at 7-day intervals until 1 month [42]. In our study, the rate of DNA presence after oral ingestion with triturated ticks was 16.66% (1/6) in mice, and 50% (3/6) in gerbils.

Gerbils are good experimental models for babesiosis caused by *B. divergens*, as they develop an acute and fatal form of the disease after intraperitoneal inoculation. Most of the clinical signs appear after 3 days pi with the animals dying after 5 days pi [43, 44]. The hepatic tissue of gerbils infected with *B. divergens* presented dilated sinusoids with macrophages, inflammation of the stroma and hyperplasia of the Kupffer cells and the spleen presented disorganization of the architecture [43–45]. Experimental and spontaneous infections of dogs with *B. canis* and *B. gibsoni* showed diffuse periportal and centrilobular hepatitis, with the presence of hemosiderin in Kupffer cells [46–48]. In dogs infected with *B. canis* histopathological changes included hepatocyte vacuolation, dilatation of hepatic sinusoids and degenerative changes, particularly in the proximal tubes of kidneys [46]. In dogs naturally infected with *B. canis* vacuolar-hydropic degeneration, especially at the level of proximal convolute tubes and also necrosis of renal tubular epithelial cells was noticed [47]. In dogs infected with *B. gibsoni*, the kidneys presented an increased number of cells in the glomeruli area [48]. All these findings are consistent with our findings, but no other evidence of infection (i.e. no *Babesia* stages) was recorded.

## Conclusions

Although the presence of DNA of *B. canis* in the tissues of rodents experimentally inoculated *via* oral ingestion with infected ticks is not a conclusive proof of the infection or the viability of the piroplasms, it still demonstrates the persistence of parasite DNA and raises further questions regarding alternative routes of transmission or controversial PCR diagnostic results.

## Data Availability

The datasets used and/or analyzed during the current study are available from the corresponding author upon reasonable request.

## Abbreviation

pi: post-infection
